# That Likeness

**Published:** 1856-04

**Authors:** 


### THAT LIKENESS.

The likeness of Dr. Drake has been the cause of some misunderstanding between the "*Counseller*" and "*Reporter.*" The difficulty seems now to lie in the terms *lithograph* and *engraving*. We would be very happy to be able to settle the point in dispute, at least for our own satisfaction, but as *we* have never seen the picture in the *Counsellor*, we cannot decide.

The "*Observer*," of Cincinnati, has a very truthful likeness of the distinguished medical pioneer.

